# Hyperadrenocorticism of calorie restriction contributes to its anti‐inflammatory action in mice

**DOI:** 10.1111/acel.12944

**Published:** 2019-04-01

**Authors:** Brian D. Allen, Chen‐Yu Liao, Jianhua Shu, Louis J. Muglia, Joseph A. Majzoub, Vivian Diaz, James F. Nelson

**Affiliations:** ^1^ Department of Cellular and Integrative Physiology University of Texas Health Science Center at San Antonio San Antonio Texas; ^2^ Barshop Institute for Longevity and Aging Studies The University of Texas Health Science Center at San Antonio San Antonio Texas; ^3^ Geriatric Research, Education and Clinical Center and Research Service South Texas Veterans Health Care System San Antonio Texas; ^4^ Department of Molecular Biology and Pharmacology Washington University School of Medicine St. Louis Missouri; ^5^ Division of Endocrinology, Department of Medicine Boston Children’s Hospital, Harvard Medical School Boston Massachusetts; ^6^ Present address: Buck Institute for Research on Aging Novato California; ^7^ Present address: Department of Pediatrics Cincinnati Children’s Hospital Medical Center and University of Cincinnati College of Medicine Cincinnati Ohio

**Keywords:** aging, calorie restriction, corticosterone, CRH, inflammation, mouse

## Abstract

Calorie restriction (CR), which lengthens lifespan in many species, is associated with moderate hyperadrenocorticism and attenuated inflammation. Given the anti‐inflammatory action of glucocorticoids, we tested the hypothesis that the hyperadrenocorticism of CR contributes to its attenuated inflammatory response. We used a corticotropin‐releasing‐hormone knockout (CRHKO) mouse, which is glucocorticoid insufficient. There were four controls groups: CRHKO mice and wild‐type (WT) littermates fed either ad libitum (AL) or CR (60% of AL food intake), and three experimental groups: (a) AL‐fed CRHKO mice given corticosterone (CORT) in their drinking water titrated to match the integrated 24‐hr plasma CORT levels of AL‐fed WT mice, (b) CR‐fed CRHKO mice given CORT to match the 24‐hr CORT levels of AL‐fed WT mice, and (c) CR‐fed CHRKO mice given CORT to match the 24‐hr CORT levels of CR‐fed WT mice. Inflammation was measured volumetrically as footpad edema induced by carrageenan injection. As previously observed, CR attenuated footpad edema in WT mice. This attenuation was significantly blocked in CORT‐deficient CR‐fed CRHKO mice. Replacement of CORT in CR‐fed CRHKO mice to the elevated levels observed in CR‐fed WT mice, but not to the levels observed in AL‐fed WT mice, restored the anti‐inflammatory effect of CR. These results indicate that the hyperadrenocorticism of CR contributes to the anti‐inflammatory action of CR, which may in turn contribute to its life‐extending actions.

## 1 INTRODUCTION, RESULTS, AND DISCUSSION

Calorie restriction (CR) is among the most robust ways to extend lifespan and delay age‐related diseases in mammals (Fontana, Partridge, & Longo, 2010; Weindruch & Walford, 1988). Considerable evidence in both invertebrates (Libert et al., 2007) and mammals (Minor, Chang, & Cabo, 2009; Park et al., 2017) indicates that cell nonautonomous factors, often driven by neuroendocrine signaling, play an essential role in mediating the life‐ and health‐span enhancing effects of CR. Less is known about the specific hormone targets of these neuroendocrine factors that ultimately promote the health‐enhancing CR state. CR lowers plasma concentrations of numerous anabolic hormones, including growth hormone (GH), insulin, and insulin‐like growth factor 1 (IGF1), which may be contributors (Masoro, 2009; Weindruch & Walford, 1988). By contrast, glucocorticoids, which play a major role in responding to stressors (Munck, Guyre, & Holbrook, 1984), are elevated in CR animals (Han, Evans, Shu, Lee, & Nelson, 2001; Klebanov, Diais, Stavinoha, Suh, & Nelson, 1995; Sabatino, Masoro, McMahan, & Kuhn, 1991), although again their roles have not be delineated.

Glucocorticoids are anti‐inflammatory (Munck et al., 1984), and attenuated inflammation is widely observed in CR animals (Frame, Hart, & Leakey, 1998; Masoro, 2009). We found, for example, that CR mice exhibited attenuated inflammation when challenged with a natural polysaccharide obtained from edible seaweeds and an inflammatory agent, carrageenan (Klebanov et al., 1995). These observations have led to the hypothesis that the hyperadrenocorticism of CR contributes to the attenuation of inflammation in CR animals (Klebanov et al., 1995; Leakey et al., 1994).

Here, we tested this hypothesis directly using a corticotropin‐releasing hormone knockout (CRHKO) mouse, which is glucocorticoid deficient (Muglia, Jacobson, Dikkes, & Majzoub, 1995) and has increased inflammation following allergen exposure (Silverman et al., 2004). We first established that the anti‐inflammatory response to CR was inhibited in these CORT‐deficient mice. We then tested whether the hyperadrenocorticism of CR was required to rescue the anti‐inflammatory status associated with CR or whether the eu‐adrenocortical state of AL mice was sufficient. This was accomplished by titrating CORT in the drinking water of CHRKO mice to match the integrated 24‐hr plasma CORT levels of AL‐fed and CR‐fed WT mice, respectively (Supporting Information Figure S1).

**Figure 1:**
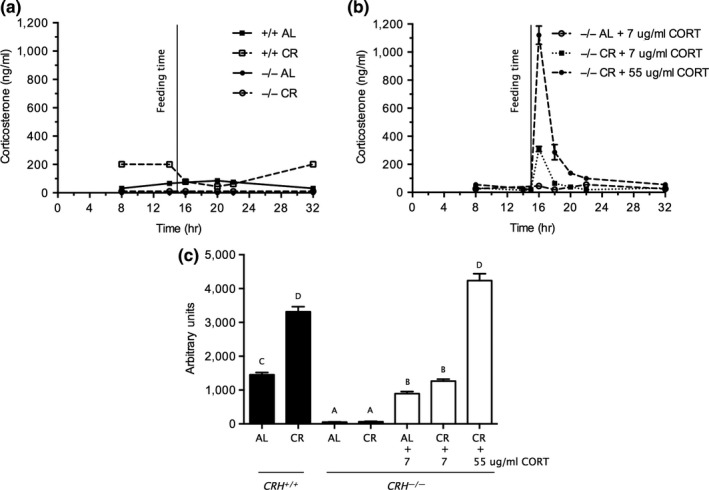
Circadian profile of plasma CORT over the 24‐hr period in WT and CRHKO mice fed AL or CR. (a) The profile from WT and CRHKO mice fed AL or CR. (b) CRHKO mice replaced with CORT in their drinking water (at 7 or 55 µg/ml) to achieve WT AL or CR levels, respectively. (c) Represents these data calculated as integrated areas under the 24‐hr curves. Data are expressed as mean ± *SE*; bars with different alphabetical letters are statistically different (*p* < 0.05 by Duncan's a posterior test). Data were analyzed by two‐way ANOVA

As previously reported (Han et al., 2001; Klebanov et al., 1995; Sabatino et al., 1991), CR markedly elevated plasma CORT in WT mice (Figure 1a,c). CORT levels integrated over the entire day were over twofold higher in CR WT than AL WT mice (*p* < 0.05, Figure 1c). In AL WT mice, CORT levels peaked during the dark phase (Figure 1a). By contrast, CORT levels in CR WT mice peaked during the light period and fell rapidly after feeding (Figure 1a). Consistent with previous studies (Muglia et al., 1995), CORT levels of AL‐fed CRHKO mice were markedly reduced (Figure 1a,c). Moreover, CR failed to increase CORT levels in CRHKO mice (Figure 1a,c). These results indicate that elevation of CORT by CR requires CRH.

Figure 1b shows the plasma CORT profiles of CRHKO mice given CORT in the drinking water to mimic CORT levels in WT AL and CR mice (7 and 55 μg/ml of CORT, respectively). Integrated 24‐hr levels of CORT in both AL and CR CRHKO mice replaced with 7 μg/ml of CORT were similar and markedly increased above vehicle‐treated CRHKO mice—approaching, albeit less than, levels of their WT counterparts (Figure 1c). Although this replacement regimen yielded a circadian fluctuation of CORT levels in CR‐fed CRHKO mice, the pattern was shifted from the diurnal elevation in CR‐fed WT mice to a sharp circadian peak tightly coupled to the time of feeding of the restricted food allotment (cf: Figure 1a,b). CR‐fed CRHKO mice given 55 μg/ml of CORT had a markedly greater postfeeding spike in plasma CORT, and the integrated 24‐hr levels were comparable to those of CR WT mice. In summary, CORT‐replaced CRHKO mice had 24‐hr integrated levels of CORT that were comparable to those of AL‐fed and CR‐fed WT mice, although the circadian patterns differed from those of WT mice.

**Figure 2:**
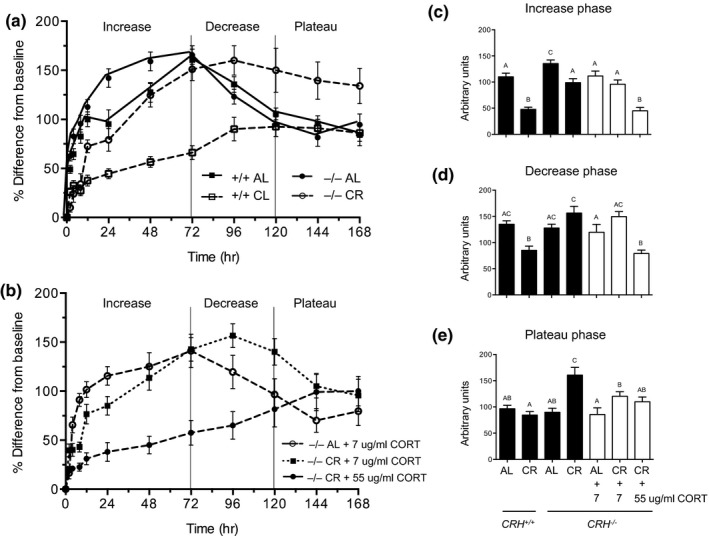
Time course of the inflammatory response to an intraplantar injection of carrageenan. (a) The responses from AL and CR WT and CHRKO mice. (b) AL and CR CHRKO mice replaced with CORT to achieve WT AL (7 µg/ml) and CR (55 µg/ml) plasma CORT levels. Edema was expressed as % difference from baseline from the increase in paw volume (ml) after carrageenan injection relative to the preinjection value for each animal (*n* = 10–12 except for CR CRHKO *n* = 6). The response (a and b) was subdivided into three phases delineating the (c) increase, (d) decrease, and (e) plateau phases of the inflammatory response in WT AL mice. The dark bars represent WT and CRHKO AL and CR mice. The light bars represent CRHKO mice CORT replaced at 7 and 55 µg/ml to mimic CORT levels in WT AL and CR mice, respectively. Data are expressed as mean ± *SE*; bars with different alphabetical letters are statistically different (*p* < 0.05 by Duncan's a posterior test). Data were analyzed by two‐way ANOVA

Using these groups, we tested the hypothesis that the CR‐mediated elevation of CORT plays a role in anti‐inflammatory action of CR, as measured by footpad edema induced by injection of carrageenan. Figure 2a shows the time course of footpad edema in the control groups (i.e., AL‐ and CR‐fed WT and CRHKO mice), and Figure 2b shows the results in the experimental groups namely, CRHKO AL and CR‐fed mice in which CORT levels were restored to levels observed in WT mice. In WT AL mice, the edematous response following carrageenan injection had three phases: first increasing to a peak at 72 hr post injection of carrageenan, then decreasing steadily thereafter until 120 hr, when it plateaued at an elevated level until measurement ceased. We analyzed the edematous responses in each of these phases (increasing, decreasing, plateau) as the integrated areas under the time‐course curves, because the responses of the experimental groups did not always vary proportionately across the three phases.

The time course of the edematous response to carrageenan during the increasing phase is shown in Figure 2a,b and the integrated areas under the time‐course curve in Figure 2c. During this phase, CR reduced edema by 60% (*p* < 0.05) in WT mice, consistent with our earlier study in WT BALB/c mice (Klebanov et al., 1995). By contrast, in CRHKO mice, CR only reduced edema by 30% (*p* < 0.05). Edema in CR‐fed CRHKO mice was more than twice that of CR WT mice (*p* < 0.05). In addition, the edema in AL‐fed CRHKO mice during this phase was 20% greater than that of AL‐fed WT mice (*p* < 0.05). CORT replacement in drinking water in CRHKO mice at 7 μg/ml, which approached levels observed in AL‐fed WT mice, rescued the edema response under AL feeding but not under CR. Only CORT replacement at 55 μg/ml, which matched the levels observed in CR‐fed WT mice, rescued the inflammatory suppression observed in AL‐fed WT mice.

The time course of the edematous response to carrageenan during the decreasing phase is shown in Figure 2a,b and the integrated areas under the time‐course curve in Figure 2d. During this phase, edema continued to be reduced by CR in WT mice (*p* < 0.05) but was not reduced by CR in CRHKO mice. In contrast to the increasing phase, CRH deficiency had no effect on edema in AL‐fed mice. CORT replacement in CRHKO mice had similar effects in the decreasing response phase to those observed during the increasing phase. Only replacement of CORT to CR‐fed WT levels reduced edema in CR‐fed CRHKO mice to that observed in CR‐fed WT mice.

The time course of the edematous response to carrageenan during the plateau phase is shown in Figure 2a,b and the integrated areas under the time‐course curve in Figure 2e. During this phase, CR did not suppress edema in WT mice. By contrast, CR exacerbated edema in CRHKO mice (*p* < 0.05), increasing it by 40% relative to both AL‐fed CRHKO mice as well as both AL‐ and CR‐fed WT controls. In contrast to the other two phases of edema, CORT replacement in CR‐fed CRHKO mice to AL WT levels (7 μg/ml) was sufficient to suppress edema, and CORT replacement to CR‐fed WT levels (55 μg/ml) had no further effect.

It should be noted that the 24‐hr patterns of plasma CORT in the CRHKO mice with CORT replacement in their drinking water (Figure 1b) differed strikingly from the profile of CORT in WT CR mice (Figure 1a) both in the magnitude of the circadian variation and the timing of the peak. These differences may reflect an intense period of drinking associated with the rapid consumption of the restricted allotment of food given the CR mice, compared to the more evenly distributed food and water intake of AL‐fed mice. Nevertheless, at least for the specific outcome of this study, the role of elevated CORT in the suppression of the edematous response to carrageenan appears to mainly result from the 24‐hr integrated levels of plasma CORT independent of the timing or magnitude of circadian variation.

The major finding of this study was that the hyperadrenocortical state associated with CR is required for the attenuation by CR of footpad edema in response to carrageenan administration in mice. This is the first direct evidence linking elevated glucocorticoids to the reduced inflammatory tone associated with CR, a well‐known characteristic of CR that is a plausible contributor to the life‐extending effects of CR.

Paradoxically, hyperadrenocorticism is well known to be detrimental to health and lifespan. In humans, chronically elevated glucocorticoids are associated with insulin resistance (Anagnostis, Athyros, Tziomalos, Karagiannis, & Mikhailidis, 2009) and are the cause of Cushing's syndrome, a life‐threatening disorder of glucocorticoid overproduction (Raff & Findling, 2003). Chronic elevation of CORT levels increases the risk of hypertension, hyperkalemia, diabetes, atherosclerosis, osteoporosis, glaucoma, and impairment of the immune and reproductive systems (McEwen & Sapolsky, 1995). Elevation of CORT damages hippocampal cells in rats (Sapolsky, Krey, & McEwen, 1985), which in turn is associated with neurodegeneration and cognitive impairment in rodents (Patel & Finch, 2002). However, all evidence for deleterious effects of hyperadrenocorticism occurs under conditions of ad libitum food intake. There is no evidence that the hyperadrenocorticism associated with CR is deleterious. The results of this study suggest that it may be beneficial—to the extent that resilience against inflammatory stressors is advantageous for the organism. These results not only suggest glucocorticoids are necessary for the anti‐inflammatory actions of CR in mice (Chung, Kim, Kim, Choi, & Yu, 2002; Klebanov et al., 1995) but also buttress previous results that hyperadrenocorticism of CR may be involved in the retardation of aging by CR (Klebanov et al., 1995; Leakey et al., 1994; Masoro, 2009; Sabatino et al., 1991).

## 2 MATERIALS AND METHODS

### 2.1 Animal husbandry

We used CRHKO mice and WT littermate controls to probe the role of hyperadrenocorticism in CR. CRHKO mice lack normal circadian variations in plasma adrenocorticotropic hormone and CORT (Muglia et al., 1995). Animals used in this study were backcrossed with C57BL/6J (B6) mice to generate a genetically defined strain. However, by the sixth backcross to B6, the null allele homozygote (i.e., CRHKO) was found to be lethal in the B6 background. We discovered that this lethality could be overcome by crossing mice carrying the null CRH allele with DBA/2J (D2) animals and breeding the resultant B6/D2 F_1_ progeny heterozygous for the CRH null allele. This strategy produced viable B6/D2 F_2 _CRHKO and WT mice that approached the expected Mendelian inheritance ratio. We used this breeding strategy with CRH null mice backcrossed into B6 to the 10th generation. The mice for this study were bred and maintained in the Aging Animal Core of The Nathan Shock Center and were born within a 3‐month period. At 4 months of age, the animals were assigned to treatment groups. All mice were fed a commercial chow (Teklad Diet LM485: Madison WI) ad libitum (AL) or calorie restricted (fed once a day at 15:00 hr). CR was introduced incrementally; mice were restricted 80% of AL the first week and 60% subsequently. AL food consumption was determined by measuring the amount of chow removed from the cage hopper and then subtracting the spillage (chow found on the cage floor). Animals were housed four per cage in a room with controlled temperature (72°F) and fixed light cycle (lights on from 06:00 to 18:00 hr). All animals had free access to water, or water plus CORT replacement at 7 μg/ml or 55 μg/ml (CORT was purchased from Steraloids Inc. Newport R.I). All procedures followed the guidelines approved by the Institutional Animal Care and Use Committee at the University of Texas Health Science Center at San Antonio and South Texas Veterans Health Care System, Audie L. Murphy Division, and are consistent with the NIH Principles for the Utilization and Care of Vertebrate Animal Used in Testing, Research and Education, the Guide for the Care and Use of Laboratory Animals and Animal Welfare Act (National Academy Press, Washington, DC).

### 2.2 Experimental design

To demonstrate the efficacy of CORT replacement in GRH KO mice, the experimental design and expected results are illustrated in Supporting Information Figure S1. This study had seven treatment groups, illustrated in Supporting Information Figure S1. There were four controls groups, WT and CRHKO littermates fed either AL or CR. Two of CRHKO groups, one fed AL and one CR, were included to determine the effect of CRH and CORT deficiency on the inflammatory response to CR. There were three experimental groups using CRHKO mice. Among these three groups, one fed AL and one CR were CORT‐replaced at 7 μg/ml drinking water to determine the effect of replacing CORT to WT AL levels on the inflammatory response. The third group was a CRHKO CR group that was CORT‐replaced at 55 μg/ml to determine the effect of CORT replacement to WT CR level on the inflammatory response.

These replacement dosages were determined from preliminary titration studies of CORT in the drinking water. All animals began CORT replacement at the commencement of CR. Group sample size was between 10 and 12 with the exception of the non‐CORT‐replaced CRHKO CR group (*n* = 6). This smaller sample size was due to deaths that were unique to this group. All mice were subjected to these feeding and CORT replacement conditions for 5 months before tested for inflammatory responsiveness.

### 2.3 Plasma collection and CORT measurements

A plasma CORT profile was obtained for each group using additional mice. Blood collected by tail bleeding at 08:00, 14:00, 16:00, 18:00, 20:00, and 22:00 hr (each animal was bled at each time point). To minimize the effect of handling stress on CORT levels, bleedings were performed at least 3 days apart and were completed within 2 min of initial disturbance of the animal. Only one animal from each cage was removed for measurement at any time. Individual animals were restrained exposing the tail where a 10mm cut was made lengthwise and blood was drawn into a 75‐mm heparinized capillary tube. Plasma was immediately prepared by centrifugation and stored at −80°C. CORT was measured using a **^125^**I corticosterone radioimmunoassay kit (ICN Pharmaceuticals, Inc. Costa Mesa, CA). The standard curves were linear (*r* **^2^** > 0.99) within a range of 25–1,000 ng/ml.

### 2.4 Chemicals

One day prior to the experiment, a 2% solution of Type IV Carrageenan (Sigma‐Aldrich) was prepared in 0.9% sterile saline allowing the compound to hydrate and solubilize. The mixture was vortexed and inverted in a 1.7‐ml micro‐tube and allowed to stand overnight at 4°C. The morning of the experiment, the solution was vigorously mixed and pulse spun before loading into a 0.5‐ml LO‐DOSE insulin syringe with a fixed 29G (0.33 × 13 mm) needle.

### 2.5 Edema induction and measurement

Footpad edema was measured using a plethysmometer (Model 7140 Ugo Basile, Italy). Edema was measured by immersing the footpad into the chamber fluid exactly to the carpel joint. The plethysmometer was calibrated before each series of measurements. The morning of the experiment, individual animals were weighed and a baseline foot measurement was recorded. The animals were restrained by rolling the upper‐half of the body in cloth leaving the hind legs exposed where the left paw was injected with 20 μl of carrageenan. The footpad was approached from the toes and injected deeply into the plantar muscle bundle to avoid leakage. All injections were completed between 07:30 and 08:00 hr. The animals were then returned to their cages until measurements at 2, 4, 8, 12, 24, 48, 72, 96, 120, 144, and 168 hr. The injected animals remained in the animal colony under routine conditions. All the feeding regimens and CORT supplementation continued to occur during the 168 hr of inflammation testing. Edema data are expressed as percent increase relative to the baseline measurement of the footpad. Each recorded measurement was the average of at least three dips. This study involved a large number of mice. Therefore, the data are the cumulative result of five sequential cohorts using two mice from each of the seven treatment groups.

### 2.6 Statistical analysis

Statistical analysis for differences among the groups was assessed by ANOVA using Statistical Package for Social Sciences (SPSS^® ^Inc., Chicago, IL) with Duncan as a post hoc test. Results are expressed as means ± *SE*, and *p* values equal to or less than 0.05 were considered to be significantly different.

## CONFLICT OF INTEREST

None declared.

## AUTHOR CONTRIBUTIONS

BDA and JFN designed the study. BDA and JS conducted the experiments. LJM and JAM provided the CRHKO mice. VD conducted the breeding, backcrossing, husbandry, and database entry and management of the mice. C‐YL and BDA performed the data analysis and wrote the manuscript with input, contributions, and comments from all authors.
