# Supplementary figures and images for: Hyperadrenocorticism of calorie restriction contributes to its anti‐inflammatory action in mice
Source: Aging Cell. 2019 Apr 1;18(3):e12944. doi: 10.1111/acel.12944 (PMC6516174; doi:10.1111/acel.12944)

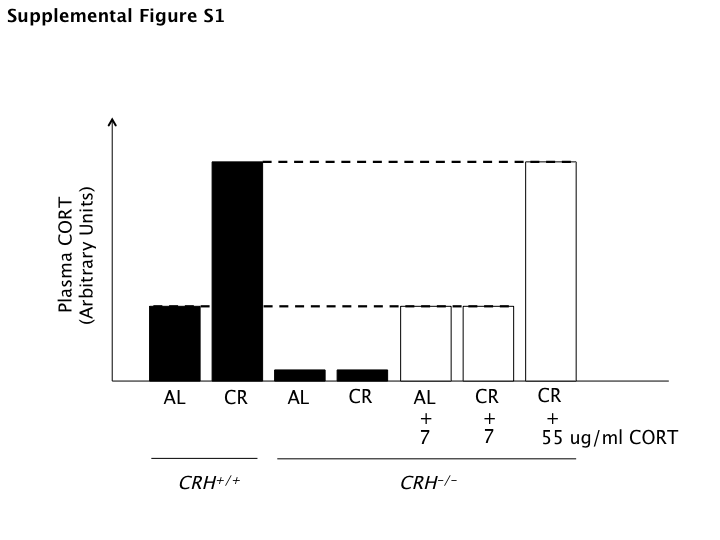

Supplement: Supplementary file 1 [file ACEL-18-e12944-s001.tiff]
